# Intraspecific genetic variation of a *Fagus sylvatica* population in a temperate forest derived from airborne imaging spectroscopy time series

**DOI:** 10.1002/ece3.6469

**Published:** 2020-06-19

**Authors:** Ewa A. Czyż, Carla Guillén Escribà, Hendrik Wulf, Andrew Tedder, Meredith C. Schuman, Fabian D. Schneider, Michael E. Schaepman

**Affiliations:** ^1^ Remote Sensing Laboratories Department of Geography University of Zürich Zürich Switzerland; ^2^ Department of Evolutionary Biology and Environmental Studies University of Zürich Zürich Switzerland; ^3^ School of Chemistry and Biosciences Faculty of Life Sciences University of Bradford Bradford UK; ^4^ Department of Chemistry University of Zürich Zürich Switzerland; ^5^ Jet Propulsion Laboratory California Institute of Technology Pasadena CA USA

**Keywords:** imaging spectroscopy, intraspecific variation, partial least squares regression, population genetics, temperate forest

## Abstract

1. The growing pace of environmental change has increased the need for large‐scale monitoring of biodiversity. Declining intraspecific genetic variation is likely a critical factor in biodiversity loss, but is especially difficult to monitor: assessments of genetic variation are commonly based on measuring allele pools, which requires sampling of individuals and extensive sample processing, limiting spatial coverage. Alternatively, imaging spectroscopy data from remote platforms may hold the potential to reveal genetic structure of populations. In this study, we investigated how differences detected in an airborne imaging spectroscopy time series correspond to genetic variation within a population of *Fagus sylvatica* under natural conditions.
2. We used multi‐annual APEX (Airborne Prism Experiment) imaging spectrometer data from a temperate forest located in the Swiss midlands (Laegern, 47°28'N, 8°21'E), along with microsatellite data from *F. sylvatica* individuals collected at the site. We identified variation in foliar reflectance independent of annual and seasonal changes which we hypothesize is more likely to correspond to stable genetic differences. We established a direct connection between the spectroscopy and genetics data by using partial least squares (PLS) regression to predict the probability of belonging to a genetic cluster from spectral data.
3. We achieved the best genetic structure prediction by using derivatives of reflectance and a subset of wavebands rather than full‐analyzed spectra. Our model indicates that spectral regions related to leaf water content, phenols, pigments, and wax composition contribute most to the ability of this approach to predict genetic structure of *F. sylvatica* population in natural conditions.
4. This study advances the use of airborne imaging spectroscopy to assess tree genetic diversity at canopy level under natural conditions, which could overcome current spatiotemporal limitations on monitoring, understanding, and preventing genetic biodiversity loss imposed by requirements for extensive in situ sampling.

## Graphical abstract

Declining intraspecific genetic variation is likely a critical factor in biodiversity loss. In this study, we demonstrated the high potential of airborne imaging spectroscopy to access information about intraspecific genetic variation in a systematic and spatially continuous manner.

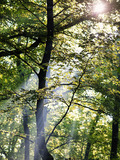

## 1 INTRODUCTION

It has long been recognized that declining genetic variation within species is a key factor in biodiversity loss (Wilson & Peter, 1988). Since then several studies have acknowledged that both inter‐ and intraspecific genetic variation have an important influence on ecosystem structure and functioning (Bolnick et al., 2011; Des Roches et al., 2018; Hughes, Inouye, Johnson, Underwood, & Vellend, 2008). A reduction in the genetic variability of a population increases its susceptibility to diseases (Schmid, 1994), limits its evolutionary potential and reduces the fitness of the next generation (Ellstrand & Antonovics, 1985). Therefore, maintaining genetically variable populations, with their wider potential range of adaptive responses, can be important for conserving biodiversity under changing environmental conditions (Gienapp, Teplitsky, Alho, Mills, & Merilä, 2008; Szathmary, Jordán, & Pál, 2001) and more frequent stochastic climatic events (Hartmann, 2013).

Considering the rapid pace of global climate change and habitat degradation in comparison with timescales of evolutionary processes, the ability of existing populations to adapt to the changes is more important than the potential emergence of new variants (Frankham, 2010). Both, the short‐term evolutionary potential and the potential phenotypic plasticity of a population are positively correlated with its allelic variation (Gratani, 2014). Therefore, the number and frequency of alleles changing in time and space is considered to be a suitable measurement of change in genetic diversity (Hoban et al., 2014).

Measurements of the allele pool, together with DNA and RNA sequencing‐based techniques (Bruford, Davies, Dulloo, Faith, & Walters, 2017; Yamasaki et al., 2017), provide direct estimates of population genetic composition, which is defined as one of the six essential biodiversity variables (EBV) for monitoring worldwide biodiversity status (Pereira, 2013). These techniques require physical sampling of individuals combined with extraction and analysis of samples (Davies et al., 2012) and are costly and time‐consuming. The resulting measurements usually lack the spatial and temporal extent relevant for biodiversity monitoring. Whereas, large‐scale monitoring is essential for understanding the drivers of biodiversity change arising from key global change at various spatial and temporal scales (Jetz et al., 2016). Continuous temporal, spatial, and spectral data derived from remote sensing platforms have the potential to overcome scale‐induced limitations and are therefore receiving increasing attention for achieving global biodiversity assessments (Navarro et al., 2017; O'Connor et al., 2015; Skidmore et al., 2015; Turner, 2014).

Both passive and active remote sensing technologies have been used to estimate functional, taxonomic, and phylogenetic diversity of plants in a variety of ecosystems. For example, Schneider et al. (2017) evaluated functional diversity in a temperate forest based on vegetation traits interpreted from imaging spectroscopy and light detection and ranging (LiDAR) data. Taxonomic diversity assessment of other sites has been conducted using both trait‐ and spectra‐based approaches (e.g., Asner & Martin, 2009; Martin, Newman, Aber, & Congalton, 1998). However, very few remote sensing studies to date have provided within‐species genetic diversity measures at a canopy level under natural conditions.

Like different species, genetically different tree individuals can express different morphological and physiological traits, which shape their reflectance features. These differences are likely to be less pronounced at lower taxonomic ranks than at the species level (Hulshof & Swenson, 2010). Therefore, the recognition of individuals of different genotypes based only on spectral information gained in nonexperimental conditions is limited and not commonly attempted. However, the increasing use of remote sensing data in ecological assessments has led to recognition of the potential for linking genetic with spectral variation. Cavender‐Bares et al. (2016) correlated spectral data from on‐leaf measurements with genetic clusters as well as species divisions for several species of oak (*Quercus*); and Schweiger et al. (2018) correlated phylogenetic relationships among grassland species with spectral information from both on‐leaf and remote (tram‐based) measurements. A remote sensing study by Madritch et al. (2014) was able to correlate foliar reflectance with genotype for quaking aspen (*Populus tremuloides*) clones under natural conditions, and furthermore revealed variation in below ground processes. Recent reviews by Bush et al. (2017) and Yamasaki et al. (2017) discussed the potential of remote sensing to reveal genetic composition in natural habitats.

Given the need for novel approaches to detect intraspecific genetic variation and the potential of using imaging spectroscopy data (Geijzendorffer et al., 2016; Navarro et al., 2017; Vihervaara et al., 2017), we attempt to identify a direct connection between the spectral and genetic information from individual trees within a temperate forest. We aim to demonstrate that genotype‐specific phenotypic features can be detected in spectral reflectance data acquired under natural conditions and at the canopy level. Furthermore, we emphasize that analyzing this spectral information could represent a time‐ and cost‐efficient tool to reveal the genetic composition of forests, repeatedly and at large spatial scales.

We base our study on the hypothesis that the foliar reflectance changes on an annual and seasonal basis, which contrasts the expression of stable, genotype‐specific phenotypic features of individual trees that are maintained over years. Based on that, we expect to identify links between remotely sensed predictors (spectral bands) and genetic structure (membership probability to the genetic clusters) which are maintained over multiple years. To test this hypothesis, we use multi‐annual airborne imaging spectroscopy data from a temperate forest in Switzerland along with genetic information derived from microsatellite analyses of individual trees at the study site. Our approach is based on the conditions that high‐fidelity spectral measurements are available (Schaepman et al., 2009, 2015), with residual measurement noise (Hueni, Damm, Kneubuehler, Schlapfer, & Schaepman, 2017) lower than the expected genetic variation to be detected; and further, that individual tree crowns (here: dominant *F. sylvatica* trees) can be detected using the spatial resolution of the imaging spectrometer, and that a minimum of 2–3 sunlit crown pixels for each individual can be identified in the airborne data. We established the link between spectral and genetic data by using partial least square (PLS) regression to assess the explanatory power of distinct wavelength regions (between 372 and 2,540 nm) within the solar radiation reflected from the tree canopy. By combining interdisciplinary approaches, we take a step toward using the potential of imaging spectrometry for spatiotemporal biodiversity mapping of genetic variation within species.

## 2 MATERIALS AND METHODS

### 2.1 Study area

**FIGURE 1:**
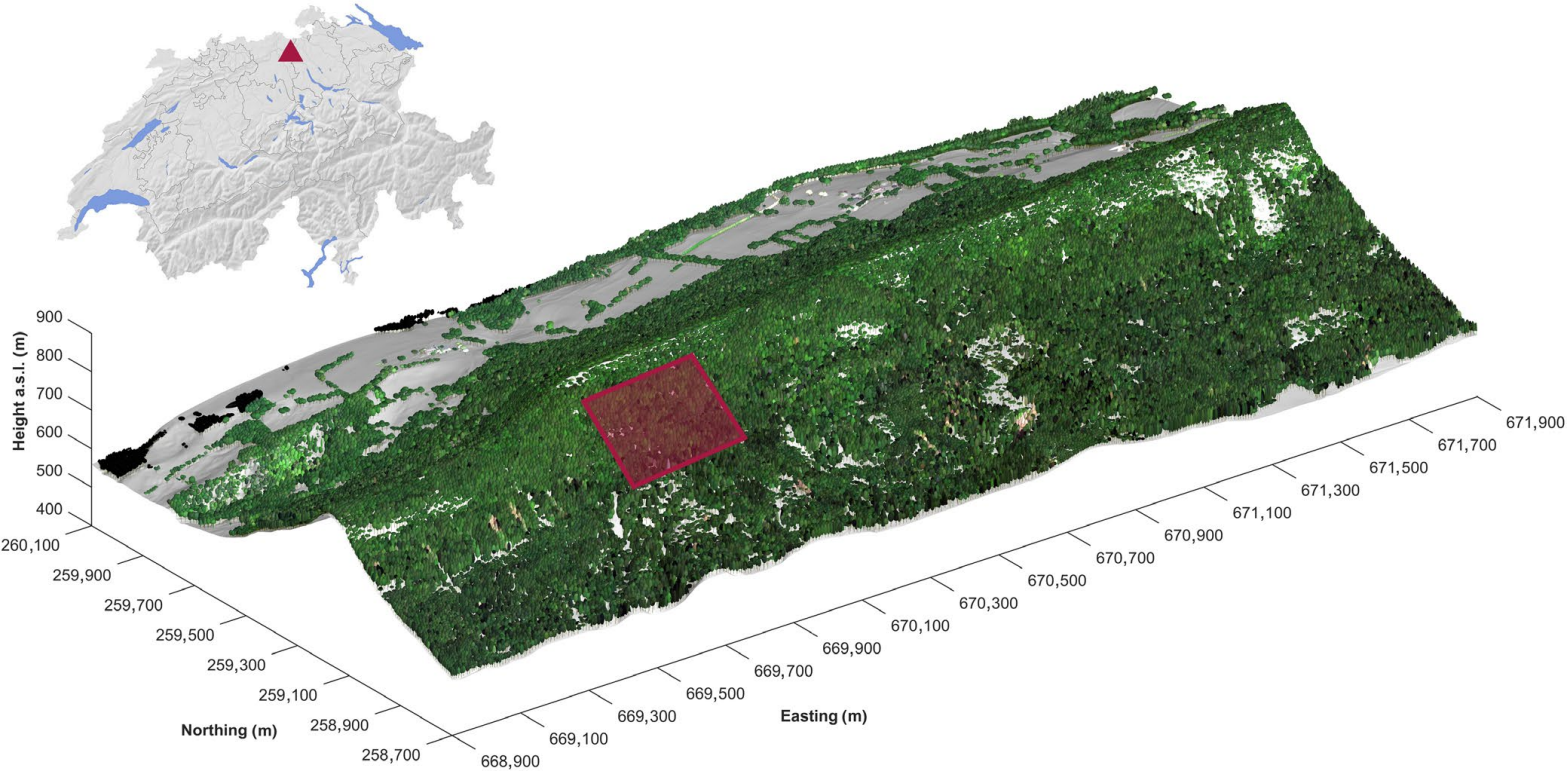
Location of the study site (rectangle). The temperate forest is located in northern Switzerland (triangle) on a portion of the south‐facing slope of the Laegern mountain

The study area covers 12.6 ha of seminatural temperate mixed forest located on the Laegern mountain on the northern boundary of the Swiss Plateau (47°28'N, 8°21'E) (Figure 1). The climate of this region is characterized by a mean annual temperature of 7.4°C and mean annual precipitation of 1,000 mm (Etzold et al., 2011). Our study site is located on an up‐to‐60°‐steep south‐facing slope with an elevation range of 620 to 810 m a.s.l (Guillén Escribà et al., inpress). According to the United Nations Environment World Conservation Monitoring Centre (UNEP‐WCMC), the vegetation cover is classified as Temperate Deciduous Broadleaf Forest, with 13 tree species consisting of 3 conifers and 10 angiosperms; the European common beech (*Fagus sylvatica)* is the dominant species. *F. sylvatica* trees are wind‐pollinated, monoecious plants. Tree age spans between 53 and 185 years with a mean height of 30.6 m and a diameter at breast height of up to 150 cm (Eugster et al., 2007). This composition creates a complex vertical structure of the mainly closed canopy (Schneider et al., 2017). The study area is located in an unmanaged part of the forest and has been a forest ecosystem research site for the last four decades (Kloeti, Keller, & Guecheva, 1989). Individual trees in the study area have been reconstructed in 3D using ground and airborne laser scanning, and modeled using 3D radiative transfer models (Schneider et al., 2014; Schneider et al. 2017), allowing to model and validate airborne data with high accuracy.

### 2.2 Genetic data

**FIGURE 2:**
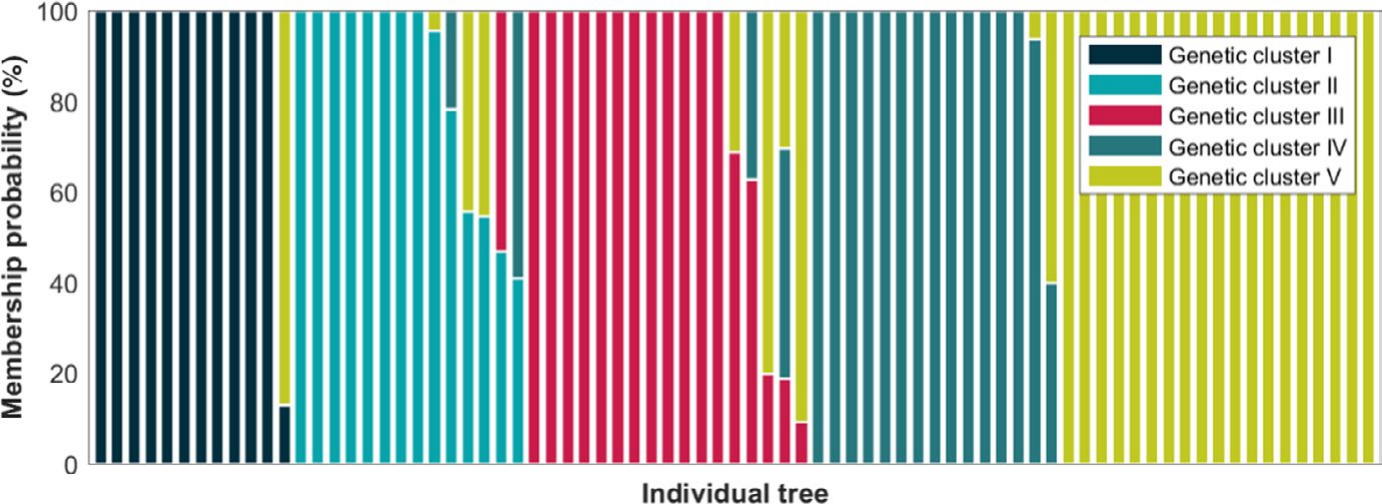
Membership probabilities for 77 sampled *Fagus sylvatica* individuals in five genetic clusters as determined by microsatellite analyses

Microsatellite genotyping was conducted on genomic DNA samples from 77 dominant *F. sylvatica* individuals located in the study area. The individuals were georeferenced using a tachymeter in April 2013. The tachymeter measurements were tied to reference points of the Swiss national grid (LV95). We used a polygon traverse and additional fixed points to measure all tree locations in the challenging terrain (Leiterer, Furrer, Schaepman, & Morsdorf, 2015). Tree crowns and tree trunks were mapped separately and linked to each other using photogrammetric approaches, due to slope geometries distorting nadir projection of crowns to trunks (Guillén Escribà et al. in press; Torabzadeh, Leiterer, Hueni, Schaepman, & Morsdorf, 2019). Genomic DNA was extracted from leaf disks (diameter 1.15 cm) sampled from each tree in September 2013. The collected material was stored on silica gel. The DNA from the sampled material was extracted using the cetyl trimethylammonium bromide (CTAB) method following the procedure of Doyle and Doyle (1990). From the extracted DNA, five highly variable microsatellite loci (FS1‐03, FS1‐15, FS3‐04, FS4‐46, FCM5; Pastorelli, et al., 2003) were amplified using Polymerase Chain Reaction (PCR). To assess the degree of polymorphism at each microsatellite locus, capillary electrophoresis was performed on an ABI‐3720 sequencer (Thermo Fisher Scientific, UK) and GeneMapper software was used to determine the length of the analyzed microsatellites for each sampled tree (Table S1). Population structure was inferred using a model‐based Bayesian clustering approach implemented in the software TESS2 (Durand, Chen, & François, 2009). Twenty independent runs were performed for K, genetic clusters; the total number of sweeps and burns was 1,200 and 200, respectively, and the degree of trend was set to linear. Based on an average cross‐entropy of the admixture model, we determined five genetic clusters, to which each tree was assigned its membership probability (Figure 2).

### 2.3 Optical data

The spectral dataset contains seven acquisitions of the Airborne Prism Experiment (APEX) Airborne Imaging Spectrometer (AIS) (Schaepman et al., 2015) acquired between 2009 and 2016. To compare trees from similar development stages, images from the range of 300 – 700 cumulative growing degree days (CGDD) (corresponding to June ‐ July) within each year were selected (Table S2). The raw APEX data preprocessing chain including calibration and correction for spectral shifts with smile effect were performed in the APEX Processing and Archiving Facility and the ATCOR smile module, respectively (Hueni et al., 2012; Hueni et al., 2009; Richter, Schlapfer, & Muller, 2011). Calibrated and corrected radiance data were atmospherically corrected to surface reflectances in ATCOR (Hueni et al., 2017; Schläpfer & Richter, 2002) resulting in imaging spectroscopy datasets of 284 spectral bands, each in the range of 372–2540 nm with 2 m spatial resolution. The wavebands most affected by atmospheric absorption features were interpolated due to high noise level. Each dataset was vicariously calibrated with repeated field spectroradiometer (ASD FieldSpec 4, Boulder, CO, USA) measurements of ground targets to ensure consistent data quality standards and cross‐compatibility of the datasets.

We geometrically coregistered the multi‐temporal APEX datasets based on a spectrally distinct landmark (i.e., Laegern flux tower). As multitemporal pixels never align perfectly, we used the spectral angle mapper (SAM) method (Kruse et al., 1993) to fill a canopy projected rectangular grid with residual abundances of the SAM to generate reflectance data within one sampling grid. Furthermore, we masked shadows as they created high spatial heterogeneity, and are affected by a lower signal‐to‐noise ratio in high spatial resolution data (Nagendra & Rocchini, 2008; Stickler & Southworth, 2008). Thus, the pixels with a cumulative reflectance less than the 30th percentile of the entire dataset were excluded from the analysis. We homogenized the tree‐specific spectral signature by adopting object‐based rather than pixel‐based analyses (Karl & Maurer, 2010). The object was defined as a tree crown for each sampled *F. sylvatica* individual; individuals were identified using images derived from light detection and ranging (LiDAR) and high‐resolution drone measurements resulting in a crown map of the site (Torabzadeh et al., 2019; Guillén‐Escribà et al. under review). The spectral signature for each *F. sylvatica* individual was a mean of sunlit pixels’ reflectance from the delineated crown. On average, 17 ± 9 pixels (mean ± standard deviation) were averaged per crown.

Using the annual APEX imagery, we based our analysis on the following three datasets: (a) yearly averaged reflectance data per tree crown, (b) *z*‐score of “a,” and (c) the 1^st^ derivative of “b.” The *z*‐score of the mean tree crown reflectance was calculated separately for each spectral band and independently for each year with the formula: (*x*_l_ − *µ*_l_)/*σ*_1_, where *x* is a value of a single spectral band from each *F. sylvatica* individual, *µ* and *σ* are respectively mean and standard deviation values of a single spectral band from the whole analyzed *F. sylvatica* population and *l* stands for spectral band. *Z*‐score of reflectance (dataset b) and their 1^st^ derivative (dataset c) were included in the analysis to reduce the impact of multi‐temporal variation in the reflectance magnitudes and simultaneously emphasize the relative differences between reflectance and absorption/transmittance influenced by structure, water content, and organic compounds (Huesca, García, Roth, Casas, & Ustin, 2016).

### 2.4 Statistical analysis

We investigated the relationship between imaging spectroscopy and genetic information by using partial least squares (PLS) regression. This method is commonly used in chemometrics (Wold, 2001) and more generally to analyze datasets that are highly collinear and have a high ratio of independent variables to observations (Wold, Ruhe, Wold, & Dunn, 1984). In comparison with multiple linear regression (MLR), the latent variables of PLS regression are generated not only based on the best explanation of dependent and independent variables, but also with respect to the relationship between them (Wold, Eriksson, Trygg, & Kettaneh, 2004). The method is used in analyzing spectral data for classification and prediction purposes (Cavender‐Bares et al., 2016; Lee et al., 2016; Peerbhay, Mutanga, & Ismail, 2013; Singh, Serbin, McNeil, Kingdon, & Townsend, 2015). In this study, for each sampled *F. sylvatica* individual, we predicted the probability of membership in one of five recognized genetic clusters rather than assigning membership, because the total genetic distance among trees in this population is relatively small. Thus, we conducted a PLS regression rather than PLS discriminant analysis (PLS‐DA).

For each *F. sylvatica* individual, we predicted membership probability to five identified genetic clusters by using the spectral fingerprint (284 spectral bands) as predictors. Due to the relationship between genetic clusters of the analyzed population, we used one model to predict membership probability in all five genetic clusters, rather than constructing separate models for each of the genetic clusters. We predicted genetic information of each analyzed tree with leave‐one‐out cross‐validation and adjusted the number of components based on the PRESS statistic (Chen, Hong, Harris, & Sharkey, 2004).

Firstly, we developed PLS models from all spectral predictors for each year separately and we calculated the variable importance of projection (VIP), by waveband. The VIP score corresponds to the contribution of each waveband in the model prediction and is dependent on variance of wavebands and the variance of genetic structure of the trees, as well as the variances between the two (Wold, 2001). We averaged derived VIP scores over 7 years.

Subsequently, we segregated all the wavebands by their importance in genetic cluster prediction. To do so, we identified local maxima of the averaged VIP scores over years and ranked them in descending order based on their prominences. We refer to prominences as the measure of how much the local maxima stands out due to its intrinsic magnitude and its location relative to other local maxima. The threshold for local maxima identification was set to 10^–5^ minimum vertical distance. We estimated the prominences using the *findpeaks* function in MATLAB (MATLAB ver. R2017b). The VIP scores that were not identified as local maxima were assumed to have lower importance than any local maxima and were assigned a ranking based on their absolute magnitude.

Afterward, we generated *N* models out of *n* spectral predictors selected based on the *n* ^th^ most prominent VIP scores, where *n* is the number of spectral variables included for each model, increasing from 1 to the total number of predictors (*N*). We calculated the root‐mean‐square error (RMSE) for each model. We developed individual models for each year separately in order to retain intra‐annual variation. We then averaged the RMSE over all seven years, which we expect should reduce the influence of phenotypic plasticity, and thus increase the influence of stable genetic variation on differences calculated from spectra.

We conducted the procedure for each of the signal transformations (datasets a, b, c under “optical data”) separately and used the *plsregress* function in MATLAB (MATLAB ver. R2017b) for model development.

We expect that the model with the lowest RMSE is constructed based on spectral information that is the most relevant to the genotype‐specific phenotypic features maintained throughout the seven years.

## 3 RESULTS

### 3.1 VIP scores

**FIGURE 3:**
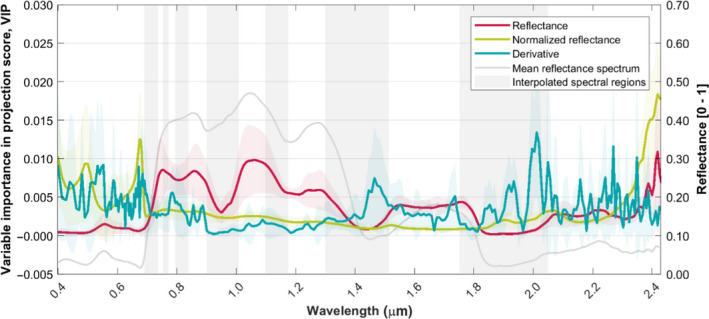
VIP scores of PLS models generated to predict genetic structure of *Fagus sylvatica* population in a temperate forest from airborne imaging spectroscopy data. The red, green, and blue solid lines represent the multi‐year mean scores achieved from analyses of reflectance (reflectance), z‐score of reflectance (normalized reflectance) and a 1^st^ derivative of z‐score of reflectance (derivative) signal transformations, respectively. The shaded areas represent the standard deviation of the scores from seven years of data acquired between 2009 and 2016. The gray bars indicate bands that have been interpolated during data processing due to high noise levels in atmospheric absorption bands, and the gray solid line represents the mean reflectance from the analyzed trees

The VIP score reveals the relevant contribution of different wavelength regions for predicting the genetic structure of sampled *F. sylvatica* individuals over the full‐analyzed solar spectrum (Figure 3).

In the reflectance dataset (dataset a, under “optical data”), the near‐infrared (0.75–1.4 μm) region of the spectrum is the most influential on predicting genetic structure. Over the full‐analyzed reflectance spectrum, the VIP score is positively correlated to the absolute reflectance (Pearson coefficient: .84, *p* < .001). Wavelengths with high VIP scores in this dataset are also characterized by higher standard deviation of interannual data than those with lower reflectance (Pearson coefficient: .86, *p* < .001).

In the normalized reflectance dataset (dataset b), the highest VIP score in predicting the genetic structure was identified for spectral features around 0.48, 0.70, and 2.40 μm. In contrast to the reflectance‐ and derivative‐based analyses, the VIP scores do not vary significantly between 0.70 and 2.30 μm and are not strongly correlated to the absolute reflectance (Pearson's coefficient: −.29, *p* < .001).

In the derivative dataset (dataset c), the spectral regions that show a closer relation to the genetic structure of analyzed trees are located at 0.55, 0.68, 1.45, 2.00, and 2.27 μm. In comparison with the VIP scores derived from reflectance and normalized reflectance datasets, the derivative dataset shows significant variation in VIP scores throughout the solar spectrum and exhibits a moderate negative correlation to the absolute reflectance (Pearson's coefficient: −.50, *p* < .001).

### 3.2 Root‐mean‐square error in prediction of the genetic structure

**FIGURE 4:**
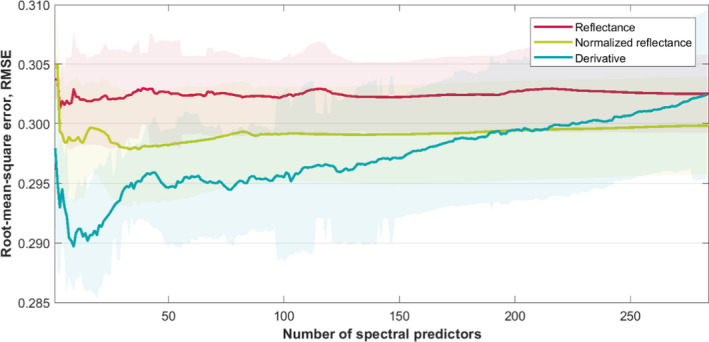
Root‐mean‐square error (RMSE) of genetic structure prediction from PLS models generated from an increasing number of most prominent (defined under “statistical analysis”) spectral predictors derived based on the VIP score of the models. The red, green, and blue solid lines represent multi‐year mean RMSE achieved from analyses made on reflectance (reflectance), z‐score of reflectance (normalized reflectance), and a 1^st^ derivative of z‐score of reflectance (derivative) signal transformations, respectively. The shaded areas represent the standard deviation of RMSE calculations derived from data acquired between 2009 and 2016

Prediction of genetic structure from the full‐analyzed spectrum resulted in higher mean RMSE than the analyses on a subset of spectral bands in all the signal transformation datasets (Figure 4). The RMSE of membership probability to the five detected genetic clusters averaged over years, trees, and genetic clusters over the full‐analyzed spectrum were 0.3025, 0.2998, and 0.3024 for reflectance (datasets a)‐, normalized reflectance (datasets b)‐, and derivative (datasets c)‐based analyses, respectively.

**FIGURE 5:**
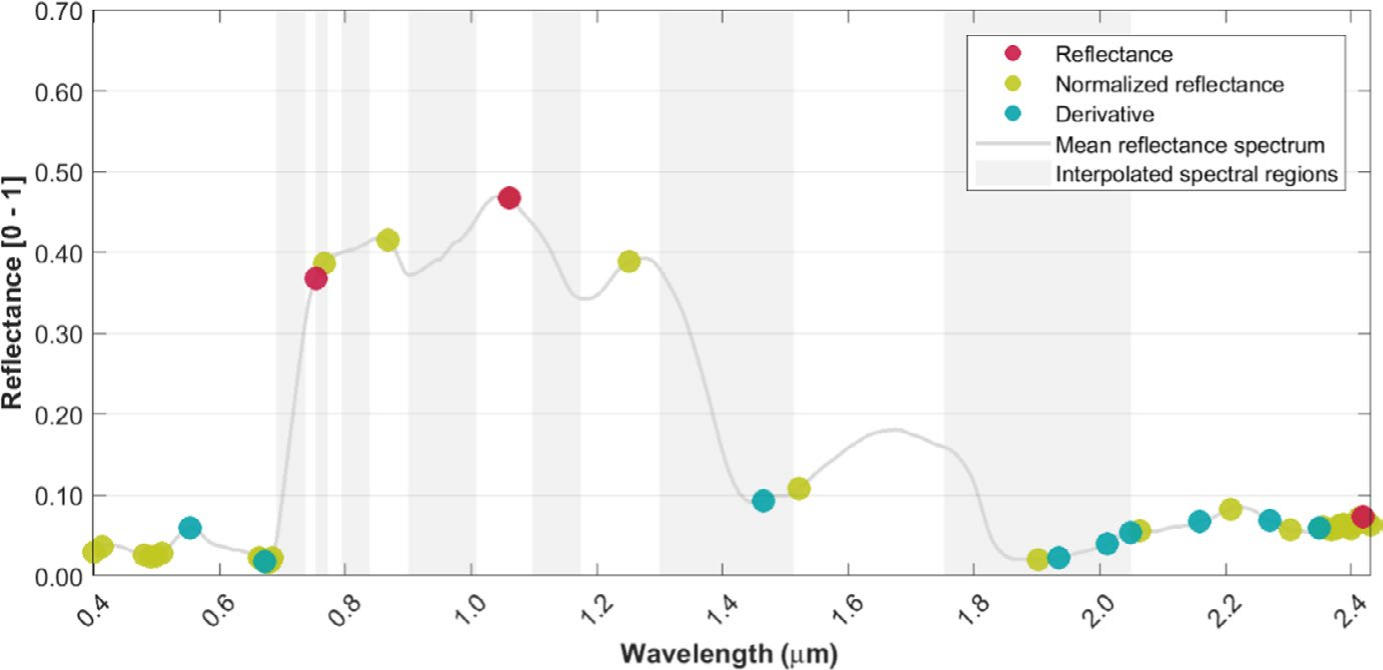
Spectral predictors selected based on the highest prominence of VIP scores (defined under “statistical analysis”), from which the genetic structure was predicted with the highest accuracy. The red, green, and blue refers to reflectance (reflectance), *z*‐score of reflectance (normalized reflectance), and a 1^st^ derivative of *z*‐score of reflectance (derivative) signal transformations, respectively. The gray bars indicate bands that have been interpolated during data processing due to high noise levels in atmospheric absorption bands, and the gray solid line represents the mean reflectance from the analyzed trees

In the reflectance‐based analyses, the use of three spectral bands with the highest prominence of the VIP scores (under “statistical analysis”) resulted in RMSE of genetic structure prediction of 0.3013. Compared with the full spectrum‐based analyses, the prediction improvement is of 0.4%. Likewise, the use of 37 spectral bands in the normalized reflectance dataset (RMSE: 0.2978) reduced RMSE by 0.7% in comparison with the model prediction based on all spectral bands. The best membership prediction was achieved for a subset of nine predictors derived from the derivative‐based signal (RMSE: 0.2897). The prediction of the model constructed from those nine variables resulted in an improvement of 4.2% in comparison with the model based on the full spectrum. In the reflectance and normalized reflectance datasets, the use of the single most prominent band did not improve the prediction. However, in the derivative dataset, the use of the single most informative predictor selected based on the VIP score, rather than the full spectrum, resulted in a 1.5% improvement in genetic structure prediction (RMSE: 0.2979). The spectral indicator subsets of the datasets that result in the best‐performing models are presented in Figure 5.

Prediction of genetic structure does not change significantly with the use of more than 120 and 90 spectral bands in reflectance and normalized reflectance datasets, respectively. In contrast, the addition of further information (i.e., additional spectral variables) in the derivative dataset has an influence on the PLS model performance. In addition to the spectral predictors that construct the models resulting in lowest RMSE, incorporation of predictors derived from 1.74, 2.24, and 2.33 μm wavelengths in the derivative and 1.55 μm wavelength in the reflectance dataset substantially reduce the RMSE of the genetic structure prediction (not shown).

## 4 DISCUSSION

### 4.1 Spectral subsets

Our results show that the use of spectral subsets rather than the full‐analyzed spectrum results in a lower error in the prediction of genetic structure.

In contrast to spectroscopic measurements made in fully controlled environments, aerial measurements under natural conditions are influenced by many factors other than the genotype‐specific features. The atmospheric influence (Richter & Schläpfer, 2002) as well as phenotypic plasticity of the trees could mask genetic variation in an electromagnetic signal acquired from airborne spectroscopy, if not correctly interpreted. Reduction of nongenetically relevant variation would result in better prediction of genetic structure. Accordingly, the use of 1%, 13%, and 3% of spectral information from the reflectance, normalized reflectance, and derivative datasets, respectively, yields the best prediction of the genetic structure in our study. Furthermore, accuracy in predicting genetic structure does not change substantially after incorporating more than 42% of reflectance‐ and 32% of normalized reflectance‐derived variables.

Despite the relatively small differences in the prediction of genetic structure for *F. sylvatica* from different spectral subsets, we present evidence that the electromagnetic spectrum of the canopy surface contains information on intraspecific genetic diversity. This conclusion is supported by the analyses of different wavelength regions having varying performance in prediction of genetic structure. The improved performance of the spectral subset‐based approach is consistent with a study by Cavender‐Bares et al. (2016), in which the bands with highest VIP scores were selected for analyses. In our study, we additionally show that the results could be improved by choosing not only the highest but also the most prominent VIP scores (defined under “statistical analysis”) derived over the spectrum (Figure S1).

Our method also reveals the spectral regions that are most appropriate for genetic structure prediction, reflecting genotype‐specific phenotypic responses, which are most consistent across years. Those regions vary in each signal dataset. In all the datasets, the most influential wavelengths are those in the short‐wavelength infrared (1.40–2.54 μm) and specifically those influenced by tissue water content (Gausman & Weidner, 1985; Tucker, 1980). The analyses may also indicate the importance of wavelengths connected with C‐H bond absorption of phenolic compounds located in this region of the spectrum (Kokaly, Asner, Ollinger, Martin, & Wessman, 2009). Additionally, in the normalized and derivative datasets, the visible region of the spectrum (0.37–0.75 μm), which is influenced by pigment amount and composition (Ustin et al., 2009) as well as epicuticular wax content (Petibon F., unpublished data), is among the most informative about genetic structure.

Our results suggest that there is intraspecific variation in water balance as well as the composition and content of phenols, pigments, and waxes for the analyzed population of *F. sylvatica*, which can be elucidated remotely using aerial imaging spectroscopy. This outcome is consistent with genetic and physiological studies. *F. sylvatica* individuals originating from different populations have provenance‐specific genetic backgrounds (Demesure, Comps, & Petit, 1996) and differences in water management (Peuke, Schraml, Hartung, & Rennenberg, 2002). Further, phenolic compounds are related to many physiological reactions of plants, including protection against UV radiation (Close & McArthur, 2002), microbial (Scalbert, 1991), fungal (Telles, Kupski, & Furlong, 2017), and herbivorous (War et al., 2012) attackers, as well as pollution (Pasqualini et al., 2003) and climatic responses (Stark, 2015). The variation in the abundance of phenols is in part under genetic control (Pereira, 2007) and is already used for remote taxonomic identification of trees (Asner, Martin, & Suhaili, 2012), and within‐species assessments. Additionally, pigments influence photosynthetic performance (Flexas, Ribas‐Carbó,, Diaz‐Espejo, Galmes, & Medrano, 2008; Flexas et al., 2012). This performance is also related to the physiological adaptation of the organism and therefore is subject to purifying selection (Arntz & Delph, 2001). Similarly, epicuticular wax content responds to environmental conditions (e.g., Schreiber, Kirsch, & Riederer, 1996) and may be genetically constrained. Correlation of the spectral features identified as being most informative about genetic structure with variation in abiotic and biotic factors may be a first step to elucidate the influence of genotype by environment interactions.

It should be noted that, due to the uncertainty of spectral measurements below 0.45 μm and above 2.20 μm, and in the spectral regions where the signal was interpolated during data processing due to high noise levels in atmospheric absorption bands (indicated by gray shading in figures), the outcomes related to those specific wavelengths should be interpreted with caution.

### 4.2 Signal transformation

The difference in outcome from analyses made on various forms of the electromagnetic signal indicate the importance of signal transformation in multi‐temporal data analyses. Our analyses indicate that the transformation of the spectrum may be particularly important in analyzing fine‐scale characteristics like intraspecific genetic variation.

The strong correlation of wavelength importance and the magnitude of the nontransformed signal could be caused by statistical limitations of the PLS regression method. The importance of specific wavelength regions might be assigned by overbalance of variation within one wavelength over fine genetically relevant information recorded at that particular wavelength. This could be supported by the observed reduction of importance of wavelengths with high magnitude in the normalized reflectance dataset, so in the signal where interannual variation caused by system instability is reduced. However, normalization may remove many sources of spectral variation, including the variation among spectral features of genetic clusters. This problem is remedied in the derivative dataset, where differences among individual measurements are emphasized and the intensity is not a major source of variation across acquisitions. Additionally, each predicting variable in the derivative dataset is an outcome of two neighboring spectral bands and thus represents more information, but also information that is averaged over a wider spectral window. The reduction of spectral resolution from high spectral resolution data results in higher signal‐to‐noise ratios (Karl & Maurer, 2010; Nagendra & Rocchini, 2008; Stickler & Southworth, 2008) and therefore may expose the spectral features for which genotype‐specific variation is greater than variation of the system acquisition.

Indeed, the derivative dataset over multi‐year analyses performed best in predicting genetic structure of the *F. sylvatica* study population, and thus, we expect this dataset conveys relatively the most information about intraspecific genetic variation derived from time series.

### 4.3 Limitations and outlook

It should be emphasized that the approach we used takes advantage of relatively high temporal, spectral and spatial resolution of available airborne data.

Using a dataset covering seven years, we have been able to reduce the influence of phenotypic responses related to interannual and phenological variability and focus on differences which remain stable across time and may be genotype‐specific. However, there are also likely to be genotype‐specific responses which are more distinct on an intra‐annual or intradaily basis. For example, investigations over the phenological cycle of plant communities (e.g., Merton, 1998) and plant species (e.g., Somers & Asner, 2014) improved genetic cluster classification. Beyond the differences in phenological responses, physiological responses over the day could be indicative of genetic differences in a population (Gallé & Feller, 2007). Therefore, we expect that multi‐seasonal and daily resolved analyses may improve the prediction of intraspecific genetic structure from spectral information.

Using other datasets lacking spectrally detailed information may not be sufficient to resolve genetic variation. At the same time, the spectral resolution we used is most certainly not sufficient to identify all genetically relevant spectral features. Next steps might be to use finer genetic resolution and to investigate various populations, anticipating a wider genetic pool that potentially expresses a larger distribution of phenotypic features accessible with the spectral resolution of current airborne sensors. Additionally, incorporation of other remotely sensed data (e.g., LiDAR (Torabzadeh et al., 2019; Valbuena et al. 2020), thermal (Ullah, Schlerf, Skidmore, & Hecker, 2012), or solar‐induced chlorophyll fluorescence (SIF) (Keller et al., 2019)) can provide means to detect additional phenotypic features indicative of genetic differences.

The 2 m spatial resolution of the airborne data and the prior mapping of the Laegern experimental forest using LiDAR data allowed us to work on canopy level in natural conditions. By incorporating structural information from ground‐based LiDAR data (Morsdorf, Kükenbrink, Schneider, Abegg, & Schaepman, 2018; Schneider, Kükenbrink, Schaepman, Schimel, & Morsdorf, 2019) as well as airborne laser scanning (Kaartinen, 2012; Schneider et al., 2014; Wang, 2016), we have been able to delineate species crowns of the area and link spectral information to *F. sylvatica* individuals. Hence, this methodological approach is expandable to other species, where individuals have a certain probability of belonging to a genetic cluster within the detection sensitivity of the imaging instrument used. RS‐based studies of genetic composition, where spatial resolution is not sufficient to recognize individual trees, might be limited to cases where the genetics of the area are highly homogenous (c.f., Madritch et al., 2014).

It should be noted that our results represent a case study limited to location and species. The available genetic information of the population was not sufficiently large to perform a spatial classification to distinct genetic clusters with high confidence using the selected genetic markers. However, we relate differences among spectral fingerprints of individuals in a population of *F. sylvatica* to genetic differences among those individuals, and we identify spectral regions and signal transformation informative about genetic structure. Using a dataset with relatively high temporal, spectral, and spatial information, we demonstrate the potential of linking spectral and genetic information. This may represent a step toward developing universal models to identify genetic variation from spectral data.

## 5 CONCLUSIONS

This study highlights the potential for multi‐temporal imaging spectroscopy data to detect intraspecific genetic variation of trees in temperate forests. We investigated the use of derivative‐based analyses and PLS‐based methods to overcome discrimination challenges caused by multi‐factorial influences on the spectral canopy signature acquired under natural conditions and at various time steps. We demonstrate that, using this approach, intraspecific genetic diversity can best be assessed using spectral subsets, rather than the full spectrum influenced by various sources. Moreover, our method successfully detected the spectral regions most indicative for genetic structure prediction, without introducing prior knowledge.

In this study, we contribute to resources available for further studies focusing on genetic diversity using remote sensing techniques. Accordingly, our results suggest that this kind of analysis, where the genetic resolution is low enough for remote detection and high enough for practical purposes, is a promising tool for tracing the landscape of genetic variation (Rocchini et al., 2010) in a direct, efficient and globally consistent way.

## CONFLICT OF INTEREST

The authors declare no conflicts of interest.

## AUTHOR CONTRIBUTIONS

**Ewa A. Czyż:** Conceptualization (equal); Data curation (lead); Formal analysis (lead); Investigation (lead); Methodology (lead); Visualization (lead); Writing‐original draft (lead); Writing‐review & editing (lead). **Carla Guillén Escribà:** Conceptualization (equal); Data curation (lead); Investigation (equal); Methodology (equal); Supervision (equal); Writing‐original draft (equal). **Hendrik Wulf:** Conceptualization (equal); Data curation (lead); Investigation (equal); Methodology (equal); Supervision (equal); Writing‐original draft (equal); Writing‐review & editing (equal). **Andrew Tedder:** Data curation (equal); Formal analysis (supporting); Investigation (supporting); Methodology (supporting); Writing‐review & editing (supporting). **Meredith C. Schuman:** Writing‐original draft (equal); Writing‐review & editing (equal). **Fabian D. Schneider:** Data curation (equal); Writing‐review & editing (equal). **Michael E. Schaepman:** Conceptualization (lead); Project administration (lead); Resources (lead); Supervision (equal); Writing‐original draft (equal).

## Supporting information

Supplementary Material

## Data Availability

Genetic variation and spectral datasets available from the Dryad Digital Repository: https://doi.org/10.5061/dryad.hqbzkh1cm.
