# Supplementary material for: Intraspecific genetic variation of a Fagus sylvatica population in a temperate forest derived from airborne imaging spectroscopy time series
Source: Ecol Evol. 2020 Jun 19;10(14):7419–30. doi: 10.1002/ece3.6469 (PMC7391319; doi:10.1002/ece3.6469)
Supplement: Supplementary file 1 — Supplementary Material [file ECE3-10-7419-s001.docx]

# SUPPLEMENT

**TABLE S1** Microsatellite length statistics for sampled *F. sylvatica* individuals derived from capillary electrophoresis using GeneMapper software.

| **Length**  **Statistic** | **FS1-03** | **FS1-15** | **FS3-04** | **FS4-46** | **FCM5** |
| --- | --- | --- | --- | --- | --- |
| standard deviation | 4.63 | 8.07 | 1.73 | 22.74 | 12.51 |
| mean | 91.55 | 111.99 | 200.95 | 251.75 | 299.87 |
| variance | 21.39 | 65.09 | 2.99 | 517.23 | 156.45 |
| maximum | 108 | 137 | 206 | 328 | 322 |
| minimum | 83 | 93 | 194 | 221 | 280 |

**TABLE S2** Date, day of the year (DOY) and Cumulative Growing Degree Days (CGDD) for the acquisition of the seven years of Airborne Prism Experiment (APEX) Airborne Imaging Spectrometer (AIS) images.

| **Date** | **Day of the year (DOY)** | **Cumulative Growing Degree Days (CGDD)** |
| --- | --- | --- |
| 17.06.2009 | 168 | 429 |
| 26.06.2010 | 177 | 368 |
| 16.06.2012 | 168 | 372 |
| 12.07.2013 | 193 | 461 |
| 18.07.2014 | 199 | 674 |
| 24.06.2015 | 175 | 438 |
| 07.07.2016 | 189 | 461 |


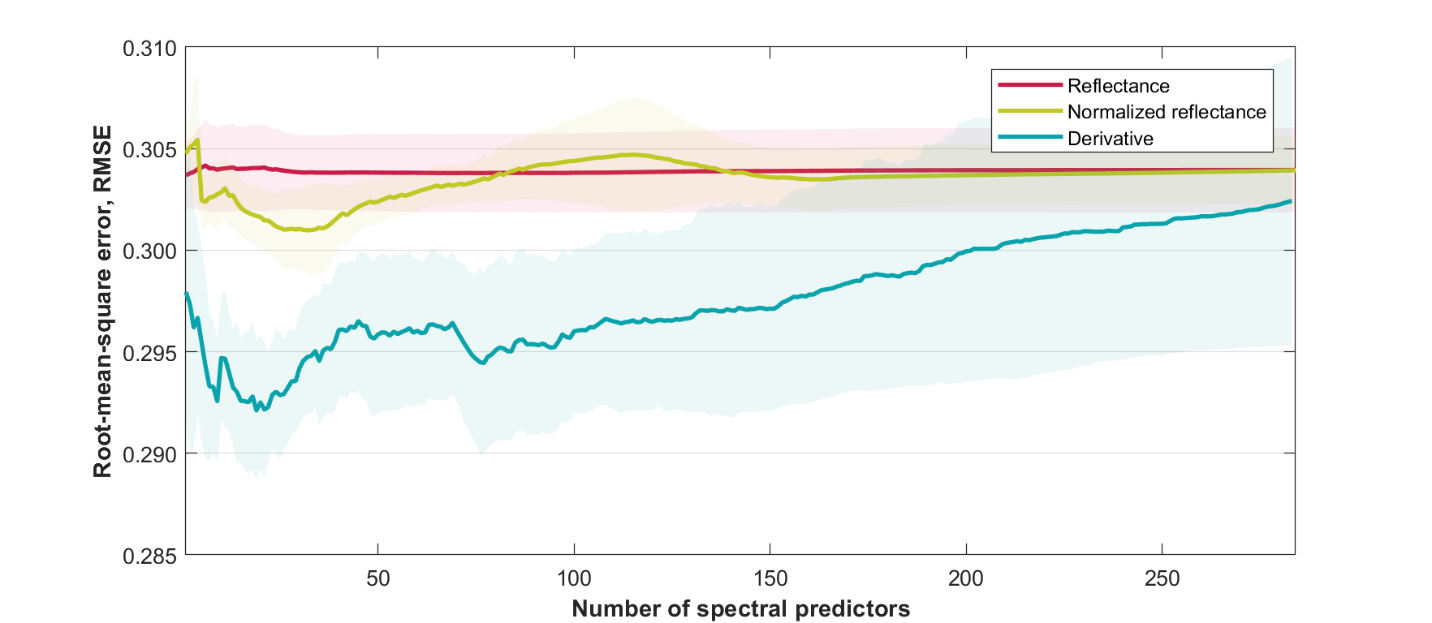


**FIGURE S1** Root-mean-square error (RMSE) of genetic structure prediction from PLS models generated from an increasing number of spectral predictors derived based on the highest VIP score of the models. The red, green and blue solid lines represent multi-year mean RMSE achieved from analyses made on reflectance (reflectance), z-score of reflectance (normalized reflectance) and a 1st derivative of z-score of reflectance (derivative) signal transformations, respectively. The shaded areas represent the standard deviation of RMSE calculations derived from data acquired between 2009 and 2016.
